# Total Knee Arthroplasty in Moderate to Severe Fixed Flexion Deformity in a Tertiary Care Center: A Descriptive Cross-sectional Study

**DOI:** 10.31729/jnma.6652

**Published:** 2021-12-31

**Authors:** Kapil Mani KC, Dirgha Raj RC, Suman Babu Marahatta, Bandhu Ram Pangeni

**Affiliations:** 1 Department of Orthopaedics, Civil Service Hospital, Minbhawan, Kathmandu, Nepal

**Keywords:** *deformity*, *flexion*, *functional status*, *ligament*, *total knee arthroplasty*

## Abstract

**Introduction:**

Performing the total knee arthroplasty in moderate to severe fixed flexion deformity, appropriate resection of bone from distal femur along with proper ligament balancing is mandatory in order to get the reasonable intraoperative correction. The aim of our study is to find out the prevalence of total knee arthroplasty among knees with moderate to severe fixed flexion deformity in a tertiary care center.

**Methods:**

This is a descriptive cross-sectional study conducted from hospital records of 2013 to 2019 in elderly patients with moderate to severe fixed flexion deformity in a Tertiary Care Hospital. Ethical clearance (14/2020) was taken from Institutional Review Board. Convenience sampling was used and statistical analyses were performed using the Statistical Package for the Social Sciences software (version 16.0). Point estimate at 95% confidence interval was calculated along with frequency and proportion for binary data.

**Results:**

Out of 400 knees with moderate to severe fixed flexion deformity, the prevalence of total knee arthroplasty was found to be 80 knees (20%) (16.08-23.92 at 95% Confidence Interval).

**Conclusions:**

The prevalence of total knee arthroplasty is comparable to other study. In our study total knee arthroplasty can be performed successfully with excellent functional outcomes in patients with moderate to severe fixed flexion deformity of knee joint provided the joint stability is maintained by appropriate ligamentous balancing.

## INTRODUCTION

Fixed flexion deformity (FFD) is commonly associated with advanced arthritis of the knee joint and it is due to the combination of ligamentous, capsular and musculotendinous contracture as well as severe bone loss from intra-articular portion of tibia and femur.^1-4^ Conservative treatment in the form of NSAIDs, physiotherapy, intra-articular injection are usually unsuccessful in severe osteoarthritis with moderate to severe FFD except Total knee arthroplasty (TKA) which gives excellent functional outcomes.^2^

Generous bone cut causes flexion instability while inadequate correction of severe flexion contracture may lead to residual flexion deformity and poor surgical results.^5-8^ Hence, it is mandatory to achieve the proper soft tissue balancing after TKA for advanced arthritis with FFD to gain satisfactory intraoperative correction, range of motion and functional recovery.^9^ It is rather recommended to limit the bone resection with generous release of posterior capsule and judicious release of ligament.^10-13^

The aim of our study is to find out the prevalence of total knee arthroplasty among kness with moderate to severe fixed flexion deformity.

## METHODS

This was a descriptive cross-sectional study based on hospital record in Civil Service Hospital from 2013 to 2019. Data was collected form hospital record (2013 to 2019) after getting permission from the institutional review board of our hospital with ethical clearance number 14/2020. Convenience sampling was used.

Sample size was calculated as

n = Z^2^ × p × q / e^2^

  = (1.96)^2^ × 0.50 × (1-0.50) / (0.05)^2^

  = 392

Where,

n= minimum required sample size
Z= 1.96 at 95% Confidence Interval (CI)
p= prevalence for maximum sample size calculation, 50%
q= 1-p
e= margin of error, 5%

Total 88 knees (66 patients) with moderate to severe FFD were treated by TKA during this period, however 6 patients (8 knees) were lost during the follow up and finally included 60 patients (80 knees) in this study. Point estimate at 95% confidence interval was calculated along with frequency and proportion for binary data.

All the patients within age range of 50 to 80 years and fixed flexion deformity of 10 to 50 degree range and those with fit for surgery were included in the study. Patients with age limit of more than 80 years and less than 50 years and FFD range of more than 50 degree and less than 10 degree were excluded from the study. Besides these, usual protocol of contraindications for TKA was followed to exclude the patients from the study. Patients were divided into the two groups (Group 1 and Group 2) with group 1 containing 29 patients (40 knees) and group 2 containing 31 patients (40 knees). The patients with fixed flexion deformity of 20 degree or less were included in the group 1 while those with FFD more than 20 degree were included in group 2. All patients were treated by posteriorly stabilized knee prosthesis of either Zimmer or Stryker depending on availability of implant and patient choice. Clinical examination of each patient and pre-operative review of radiographs were performed before surgical intervention.

Under all aseptic precautions with application of tourniquet, anterior midline skin incision followed by standard medial parapatellar incision was given to expose the knee joint. With the use of intramedullary femoral and extramedullary tibial alignment rod, measured resection of both tibia and femur was performed. Measured resection implies cutting of bone from intra-articular portion of knee joint perpendicular to mechanical axis and replacing by the implant of same thickness of removed bone. After achieving the coronal plane ligament balance, osteophytes on posterior aspect of both tibia and more importantly femur were removed followed by generous release of capsule from posterior aspect of femur with the use of curve osteotome. Special care was taken not to release too much ligament which otherwise cause severe flexion instability afterwards. In severe flexion deformity (group 2 patients), it is sometimes mandatory to release medio-lateral corner along with the contracted medial and lateral head of gastrocnemius as well as to cut extra 2 to 3 mm distal femur so that equal flexion and extension gap maintaining the reasonable joint tension was secured. Even though achieving the full extension for severe flexion deformity at the end of surgery is a primary goal, there should be high threshold to remove the extra bone from the distal femur which may otherwise cause the severe flexion instability.

All patients were given low molecular weight heparin for prophylaxis of deep vein thrombosis for 5 days post-operatively, first dose of which was started 12 hours after surgery. Second generation cephalosporin was given intravenously within half an hour of surgery preoperatively and continued for three days postoperatively. The postoperative radiographs of knee joint both antero-posterior (AP) and lateral view was done on the same day of surgery . The same postoperative protocol was followed for both groups of patients, which included walking with the help of crutches, active and passive mobilization of knee joint, isometric quadriceps and hamstring strengthening exercises after second postoperative day. The most important aspect was full time application of long knee brace for first five days after surgery in both groups of patients and then applied at night time only for another one month. Patients were followed up in Outpatient Department (OPD) 2 weeks, three months, six months and yearly after that. During each visit, clinical assessment was performed in order to calculate fixed flexion deformity and Knee Society Score (KSS) along with knee radiograph to assess the stability and any radiolucency around the implants. Statistical analyses were performed using the Statistical Package for the Social Sciences software (version 16.0). Point estimate at 95% confidence interval was calculated along with frequency and proportion for binary data.

## RESULTS

Out of 400 knees with moderate to severe fixed flexion deformity, the prevalence of total knee arthroplasty was found to be 80 knees (20%) (16.08-23.92 at 95% Confidence Interval). The demographic profiles of the patients were demonstrated in (Table 1).

**Table 1:** Showing the demographic profiles of both groups.

Demographic parameters	Group 1 FFD <20 degree	Group 2 FFD >20 degree
Age (years)	63.80±5.61	66.42±5.69
Duration of symptoms	7.68years	11.67years
	n (%)	n (%)
Difficulty in walking	12 (36.6)	21 (70)
Associated crepitus	14 (46.6)	19 (63.3)

Preoperative and postoperative FFD were mentioned in (Table 2).

**Table 2:** Showing the preoperative and postoperative fixed flexion deformity.

Parameters	Group 1 FFD <20 degree	Group 2 FFD >20 degree
Preoperative FFD	16.27±2.89	30.37±5.02
Intraaoperative FFD	0.32±1.07	1.35±1.98
FFD (2 weeks)	4.50±2.08	9.15±3.28
FFD (6 months)	1.82±1.50	4.17±2.99
FFD (1 year)	1.57±1.29	3.70±2.42
FFD (final follow up)	1.60±1.21	3.60±2.28

Average follow up duration was 4.45±0.96 (2 to 6) years.

## DISCUSSION

Long term course of chronic diseases like osteoarthritis and rheumatoid arthritis (RA) is usually associated with flexion contracture of knee joint because of inflammatory process in RA and osteophytes formation mainly on posterior aspect of distal femur in case of degenerative or traumatic OA. Severe FFD of knee joint is frequently accompanied by the posterior subluxation of tibia, valgus and external rotation deformity which is further exaggerated by contracture of iliotibial band and biceps femoris tendon.^15^ The success of TKA in FFD of knee depends on a number of factors, the most important of which are preoperative conditions of knee joint, surgical technique and degree of intraoperative correction and postoperative physiotherapy.^16^ Kenneth Cheng, et al. demonstrated that patients with a preoperative fixed flexion deformity show continued improvement in their fixed flexion up to ten years post arthroplasty and have similar outcomes to those with no preoperative fixed flexion.^17^ Randomized clinical trial of Papotto BA demonstrated that use of dynamic extension splints during night time reduces the severity of flexion deformity after TKA.^16^

Various steps for addressing the flexion deformity during TKA have been mentioned which include ligamentous release, additional resection of distal femoral bone and use of constrained prosthesis.^11^ Appropriate soft tissue balancing with generous release of posterior capsule, controlled release of ligaments and judicious resection of distal femur are key steps for success of TKA which not only achieves the obvious correction of deformity intra-operatively but also significantly improve the range of motion and functional results.^7,11^ Appropriate soft tissue balancing can be assessed intra-operatively by doing the varus and valgus stress test after the prosthesis was implanted with joint laxity not more than 2mm.^18^

In the current study average age of patients in group 1 was 63.80±5.61 years and in group 2 was 66.42±5.69 years with mean preoperative FFD of 16.27 ±2.89° in group 1 and 30.37 ±5.02° in group 2. Since the functional results in too elderly patients were guarded, we excluded the patients more than 80 years. Fixed flexion deformity more than 50° could be associated with guarded functional results and intraoperative peroneal nerve palsy. Likewise, knees with FFD of 10 degree or less do not have difficult approach to correct the deformity in comparison to normal knee. Therefore, flexion contracture more than 50 degree and less than or equal to 10 degree were excluded from the study.

In our study, mean preoperative FFD in group 1 versus group 2 was 16.27±2.89° versus 30.37±5.02°. Similarly, mean FFD in group 1 versus group 2 at the time of surgery, 2 weeks, six month, one year and at final follow up were 0.32±1.07 versus 1.35±1.98°, 4.50±2.08 versus 9.15±3.28°, 1.82±1.50 versus 4.17± 2.99°, 1.57±1.29 versus 3.70±2.42° (P value <0.001) and 1.60 ±1.21 versus 3.60±2.28° respectively. The result shows that there was near complete correction of flexion deformity intra-operatively in group 1 which even though slightly increased in two weeks, but resumed to near normal level at final follow up gradually with disciplined postoperative rehabilitation. Meanwhile we are not able to get the complete correction of flexion deformity in group 2 at the time of surgery which obviously increased at two weeks but gradually improved at final follow up, however full correction was not possible. Regarding the Knee Society Score, mean improvement of score in group 1 versus group 2 was from 46.72±7.78 versus 36.82±7.80 to 90.32±4.12 versus 87.52±5.54 at final follow up. Just like TKA for other conditions, KSS was increased significantly from preoperative to final follow up stage in both groups, however there was more improvement in group 1 in comparison to group 2.

McPherson, et al. in their series of 29 knees treated with TKA for flexion deformity, reported improved range of motion with average postoperative flexion contracture of 10.5° decreased to 2° at 24 months follow up.^19^ Firestone, et al. in their study of 40 knees described that preoperative flexion deformity greater than 20° was reduced to 3.1° immediately after surgery which increased to 10.1° at 3 months and again decreased to 7° at 2 years postoperatively.^20^ Even though the authors resected up to 5mm of extra distal femur to achieve the intraoperative correction, they did not noticed instability, decreased quadriceps strength and extensor lag. However, Tanzer and Miller had mentioned the opposite view regarding the over resection of distal femur because of instability and abnormal kinematics of joint.^21^ In the current study, we did not do over resection of distal femur up to or more than 5mm. Routine 2mm additional resection of distal femur was performed in severe flexion contracture in group 2 patients, while it was not even needed in group 1 patients with less than 20° FFD.

Regarding the optimal results of TKA in FFD of knee joint, one of the important aspects is addressing the posterior cruciate ligament (PCL). In spite of our routine use of posteriorly stabilized (PS) prosthesis in this study, there are several studies regarding the use of cruciate retaining (CR) prosthesis with varying results. The previous study of Laskin, et al.^22^ reported average postoperative flexion contracture of 11° in patients treated with CR design and preoperative FFD greater than 10 to 15°, however the study of Berend KR, et al.^11^ had reported 97% successful results in CR knee when the deformity was corrected intra-operatively without ligamentous instability. Lombardi, et al.^23^ mentioned that the treatment of patients having primary total knee arthroplasty, consistently provides excellent clinical results either retaining or sacrificing the posterior cruciate ligament.

We have followed the operative algorithm for correction of flexion deformity in TKA using PS design prosthesis which is modified from the approach given by Berend KR, et al.^11^ for both CR and PS design.

Lack of exclusion of other comorbid diseases in both groups of patients is major limiting factor of this study. Since the study is not totally blinded to both surgeons and patients, some degree of biasness is unavoidable.

## CONCLUSIONS

The prevalence of total knee arthroplasty is comparable to other study. TKA can be performed successfully in patients with moderate to severe fixed flexion deformity of knee joint provided the joint stability is maintained by appropriate ligamentous balancing. A gradual improvement in the FFD can be expected up to few years after surgery and a small residual flexion contracture does not cause functional deficit.
